# Printing and Folding: A Solution for High-Throughput Processing of Organic Thin-Film Thermoelectric Devices

**DOI:** 10.3390/s18040989

**Published:** 2018-03-27

**Authors:** Seyedmohammad Mortazavinatanzi, Alireza Rezaniakolaei, Lasse Rosendahl

**Affiliations:** Department of Energy Technology, Aalborg University, Pontoppidanstraede 111, DK-9220 Aalborg, Denmark; lar@et.aau.dk

**Keywords:** flexible thermoelectric generator, body sensor, organic thermoelectric (OTE), multiphysics simulation, wearable electronics, printed electronics

## Abstract

Wearable electronics are rapidly expanding, especially in applications like health monitoring through medical sensors and body area networks (BANs). Thermoelectric generators (TEGs) have been the main candidate among the different types of energy harvesting methods for body-mounted or even implantable sensors. Introducing new semiconductor materials like organic thermoelectric materials and advancing manufacturing techniques are paving the way to overcome the barriers associated with the bulky and inflexible nature of the common TEGs and are making it possible to fabricate flexible and biocompatible modules. Yet, the lower efficiency of these materials in comparison with bulk-inorganic counterparts as well as applying them mostly in the form of thin layers on flexible substrates limits their applications. This research aims to improve the functionality of thin and flexible organic thermoelectric generators (OTEs) by utilizing a novel design concept inspired by origami. The effects of critical geometric parameters are investigated using COMSOL Multiphysics to further prove the concept of printing and folding as an approach for the system level optimization of printed thin film TEGs.

## 1. Introduction

**Figure 1:**
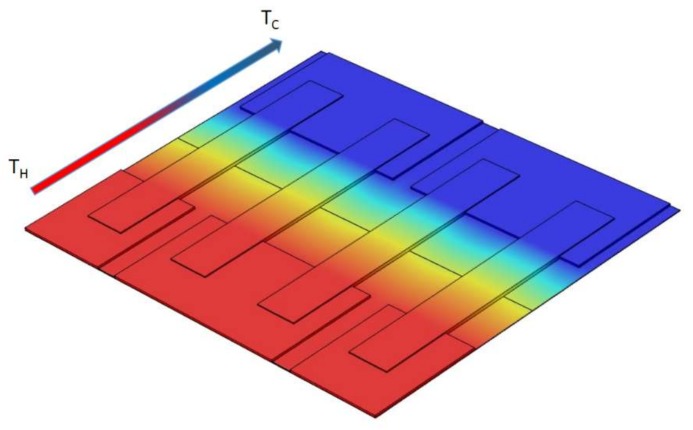
Lateral heat transfer configuration for printed thin-film thermoelectric generators (TEGs).

There is a considerable amount of energy generated by the human body in the form of biomechanical and thermal energies, but most of it is usually wasted into the surrounding environment. There are significant potentials to harvest some of these wasted energies by means of devices, which benefits from the physical effects such as piezoelectricity [1], triboelectricity [2], and thermoelectricity [3]. Among these effects, thermoelectricity has abundant advantages to power wearable electronics, since it makes it possible to produce electricity from body heat as a permanent source of energy and in a reliable manner, rooted in the solid-state nature of this effect [4]. The devices fabricated based on this concept are able to convert the steady flow of heat generated by the temperature differential between the skin and the ambient into the electricity through the Seebeck effect. There have been many attempts in recent years to take the advantage of this effect in order to build an efficient thermoelectric generator (TEG). They succeed to reach hundreds of µW of power, which enables us to make a wide range of self-powered body sensors [5,6,7,8,9,10,11,12,13,14,15,16]. In order to use the TEG as an ergonomic in-situ energy harvester, it should be flexible to enhance heat transfer from the body to the TEG and to prevent the use of multiple connected rigid TEGs [16]. Most of the attempts at body heat harvesting consist of TEGs fabricated from bulk thermoelectric material [5,6,7,9,10,16]. Despite all the advantages that these materials pose, there are major limitations for applying them in commercial applications. As an example, fabricating a flexible module is hard to achieve through this material, and they are not suitable for mass production. This makes the other types of materials including both organic and inorganic and manufacturing methods attractive (sputtering and evaporation [17,18,19], electrodeposition [20,21]m and material printings) for fabricating commercial and flexible TEGs. Among these manufacturing methods, printing technology such as inkjet printing [22,23,24], screen-printing [25,26,27], dispenser printing [28,29], and 3D printing [30,31,32] enables the opportunities for examining a diverse range of materials for building flexible TEGs in a large production scale. Despite the solution processability brought by these printing methods, as TEGs are fabricated mostly in a thin planar shape, the resulting thermoelectric legs and the temperature gradient across them are very small and can produce only a limited output voltage. Hence, most of the designs are based on an in-plane architecture (Figure 1) to take the advantage of carrier transport in the plane of the flexible substrate [33]. Although considering this concept makes organic materials easier to process from the solution it dictates a major restriction due to the fact that, in most cases, like body heat harvesting, a great portion of the thermal gradient happens across the TEGs. Consequently, device configuration and system level optimization, which have been rarely studied by the previous researchers, become important to fill this gap. For example, by using specific geometries for device architecture, it is possible to have both module cross plane heat transfer and in-plane heat transfer through the thermoelectric active materials at the same time [34,35,36].

In this work, an origami-like concept is used to design a planar flexible TEG in order to increase the thermal gradient caused by the body warmth through the module cross plane. Then, the proposed configuration is modeled in COMSOL Multiphysics, version 5.3 (supported by COMSOL A/S, Lyngby, Denmark) based on the state of the art characteristics of organic thermoelectric materials. The module configuration is optimized with regard to the thermoelectric active material geometry.

## 2. A TEG on the Human Body

The voltage on a typical thermocouple consists of *n* pairs of *p*- and *n*- type legs with the Seebeck coefficient of *S_p_* and *S_n_* for *p*- and *n*- type legs, respectively, and thermal gradient of *ΔT* between the hot and cold sides of the legs, which can be calculated as

(1) $$V_{o}=n\;\left(s_{p}+\;s_{n}\right)\;\Delta T$$

**Figure 2:**
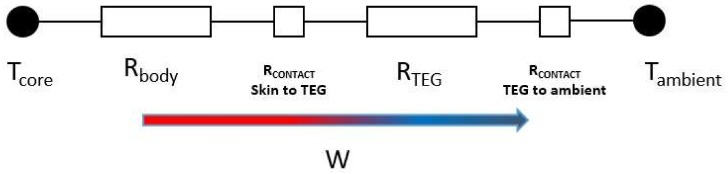
Thermal equivalent circuit of a TEG on human skin.

To effectively harvest body warmth, it is necessary to keep this thermal gradient *ΔT* at the highest possible amount. There are many parasitic thermal losses in the way of the heat transfer from the body core to the ambient, which makes it difficult to obtain the highest amount of the thermal gradient. Figure 2 shows a simplified thermal circuit of a TEG on the human skin. In addition to the thermoelectric thermal resistance, there are also obstacles like skin resistance between the body core and the skin surface. Besides, the skin contact resistance caused by the poor surface quality of human skin leads to dissipate more of the heat current. These limitations need to be addressed in a way that the mounted TEG on the body would be able to make enough power despite the small amount of feasible thermal gradient. Based on Equation (1), increasing the number of thermoelectric legs in a TEG produce more voltage. With this regard, considering the bismuth telluride as the most commonly used material for the thermoelectric generators, to produce an electrical voltage in the order of 1 V, thousands of these legs are required, which leads to increasing the total size of the generator and 5h3 complexity of the module fabrication regarding miniaturization.

On the other hand, utilizing a typical inorganic thermoelectric material like bismuth telluride for low-grade energy harvesting such as human body warmth forces us to use a heat exchanger since these materials have relatively high thermal conductivity, which makes it difficult to maintain the temperature gradient across the device. Consequently, adding a heat exchanger increases the total size and cost of the device. Organic thermoelectris can be an alternative, as the lower thermal conductivity in these conducting polymer–based materials results in a uniform thermal gradient distribution across the device. Besides, these materials are cheap and solution processable, which makes them suitable for fabricating the TEGs cheaply and quickly. The possibility of tailoring the state-of-the-art manufacturing method such as the additive manufacturing also brings the opportunity of doing the miniaturization and mass production at the same time. Despite all these advantages, organic materials have a significantly lower Seebeck coefficient compared with the inorganics counterpart and are normally processed in thin film architecture, which leads to lower efficiency and requires it to be retrieved through the device design and architecture optimization. In this study, we tried to address the low thermal gradient in the printed thin film devices through device geometry modification, which can be considered as a part of the TEGs’ system level optimization.

## 3. Printing and Folding of Thin Film Legs

**Figure 3:**
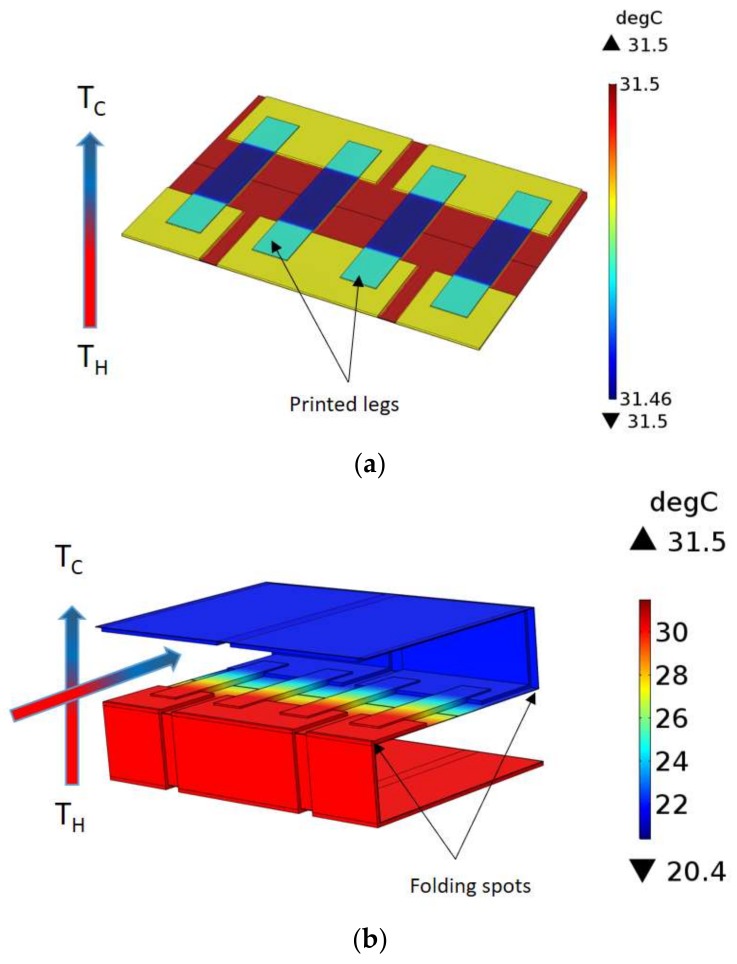
Folding and its effect on the total thermal distribution—(**a**) flat TEG, (**b**) folded TEG.

All utilized additive manufacturing methods deposited the thermoelectric active material with a thickness of tens of microns to fabricate the thermocouples. This limitation makes the use of in-plane thermal gradient (Figure 1) and lateral configuration a must for the device architecture, as the thin layer of active material is not capable of producing a desirable amount of electricity. On the other hand, in practice, the thermal gradient that happens through the device cross plane direction (Figure 3a) is small. This limitation can be addressed through the module design in a way that both the thermocouple in-plane and module cross plane heat transfer happen at the same time. In this work, an origami-like concept is developed to fold a flexible substrate and conduct the heat in the desired direction (Figure 3b). This also leads to an increase the thermal gradient between the two ends of each thermoelectric leg, consequently harvesting more electricity.

As shown in Figure 3, the small amount of thermal gradient in case “a” (less than 1 °C), which was generated by placing the printed TEG directly on the body, can be boosted to almost 12 °C after folding in case “b”. Based on this approach, organic thermoelectric materials are deposited in predefined patterns on a flexible substrate by means of a printing technique like dispenser printing or screen printing, and then the flexible substrate is folded to the final configuration. This two-step manufacturing concept—print and fold—can even be further tailored to the high throughput fabrication methods like roll-to-roll manufacturing and eventually decrease the cost and time of the production.

## 4. Design and Multi-Physics Simulation

**Figure 4:**
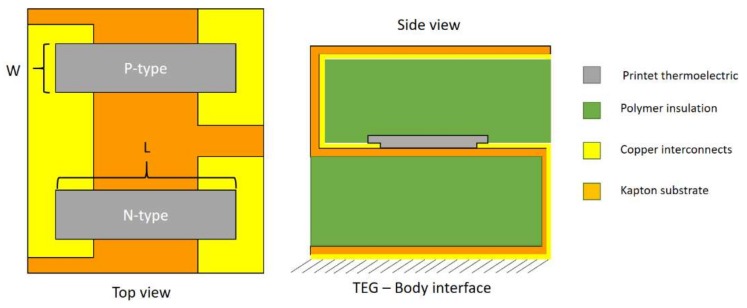
Device configuration and printed thermocouples-interconnects arrangements.

Figure 4 shows the configuration of the printed thermoelectric legs and copper interconnects on the kapton substrate. This configuration leads to a desired number of thermoelectric legs connected electrically in series and printed on the flexible substrate. T_l_, L, and W are the thermoelectric legs thickness, length, and width, respectively, and other geometrical parameters of the devices are shown in Table 1. These amounts are tunable for further optimization in order to find the optimum performance of the device.

The multi-physics simulation in this work is carried out through the thermoelectric section of the COMSOL software package, version 5.3 (supported by COMSOL A/S, Lyngby, Denmark). Both the temperature variation, *T*, and voltage, *V*, are calculated using the default governing equation in this section. The following are the differential equations utilized to model the TEG:

(2) $$-\nabla \;\left[\left(\sigma s^{2}T+K\right)\right]\nabla T-\nabla \left(\sigma s\nabla V\right)=\sigma \left[{\left(\nabla \right)}^{2}+s\nabla T\nabla V\right]$$

and

(3) ∇(σS∇T)+∇(σ∇V)=0

where *s* is Seebeck coefficient, *K* is the thermal conductivity, and *σ* is the electrical conductivity, respectively. In this work, the conducting polymer (PEDOT: Tos + TDAE) [22] is used as the p- type thermoelectric material, while the n-type material is an imaginary material with identical properties. The input parameters applied to this model are illustrated in Table 1 including the material properties, device basic geometries and physical boundary conditions. 

**Figure 5:**
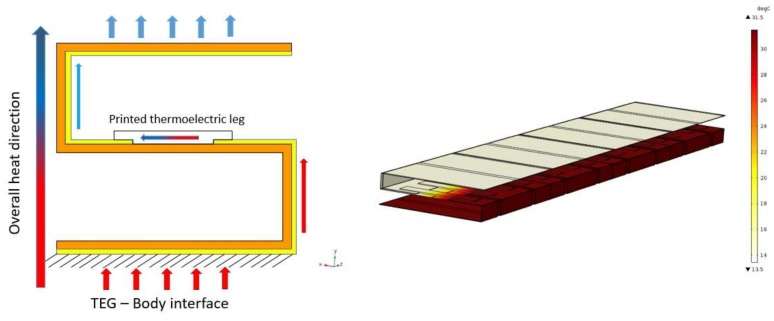
Thermal distribution after mounting the device on the human body.

Figure 5 shows the thermal distribution and heat flow direction of the device placed on the body. As stated in Table 1, the natural convection at the cold side of the TEG is 5.46 W/m^2^-K, which is calculated based on the ANSI/ASHRAE Standard 55 [37] and as a function of the air velocity. The heat rejected from the cold side without the need for bulky heat sinks used in conventional TEG systems. The effect of thermal contact resistance at the skin-TEG interface is not considered in this study since the focus is mainly on the evaluating of the effect of TEG geometrical features on the output power.

## 5. Analysis

**Figure 6:**
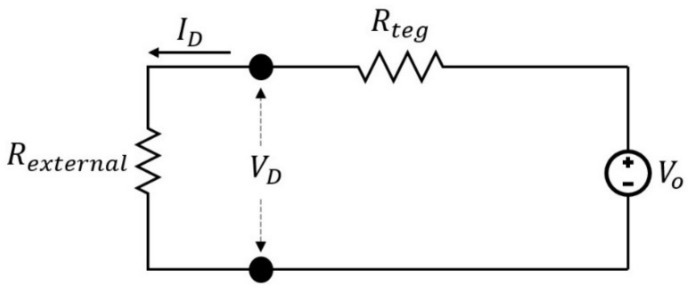
Electrical equivalent circuit.

In this section, it is shown that the proposed device geometry can be further optimized by altering the different parameters and a finite element method (FEM) tool like COMSOL Multiphysics version 5.3—supported by COMSOL A/S, Lyngby, Denmark. The comparison between the alternative situations is carried out based on the produced maximum output power in the form of voltage-current-power curves. This maximum power is basically a function of the TEG’s length, width, thickness, and the amount of the total thermal gradient across the device. It normally occurs when the device electrical resistance matches the external load resistance, based on the maximum power transfer theorem. Figure 6 shows the electrical equivalent circuit of the TEG.

Where *V_D_* is the produced electrical potential by the device, which makes the electrical current of *I_D_* flows through the external load of *R_external_*. *R_teg_* is the overall electrical resistance of the TEG, and *V_O_* is the open circuit voltage. The produced power is calculated as follows:

(4) $$P_{D}=\;V_{D}\;I_{D}=\;\frac{V_{D}{}^{2}}{R_{external}}=\;\frac{{\left[\;\frac{V_{O}\;R_{external}\;}{R_{external}+\;R_{teg}\;}\;\right]}^{2}}{R_{external}}$$

**Figure 7:**
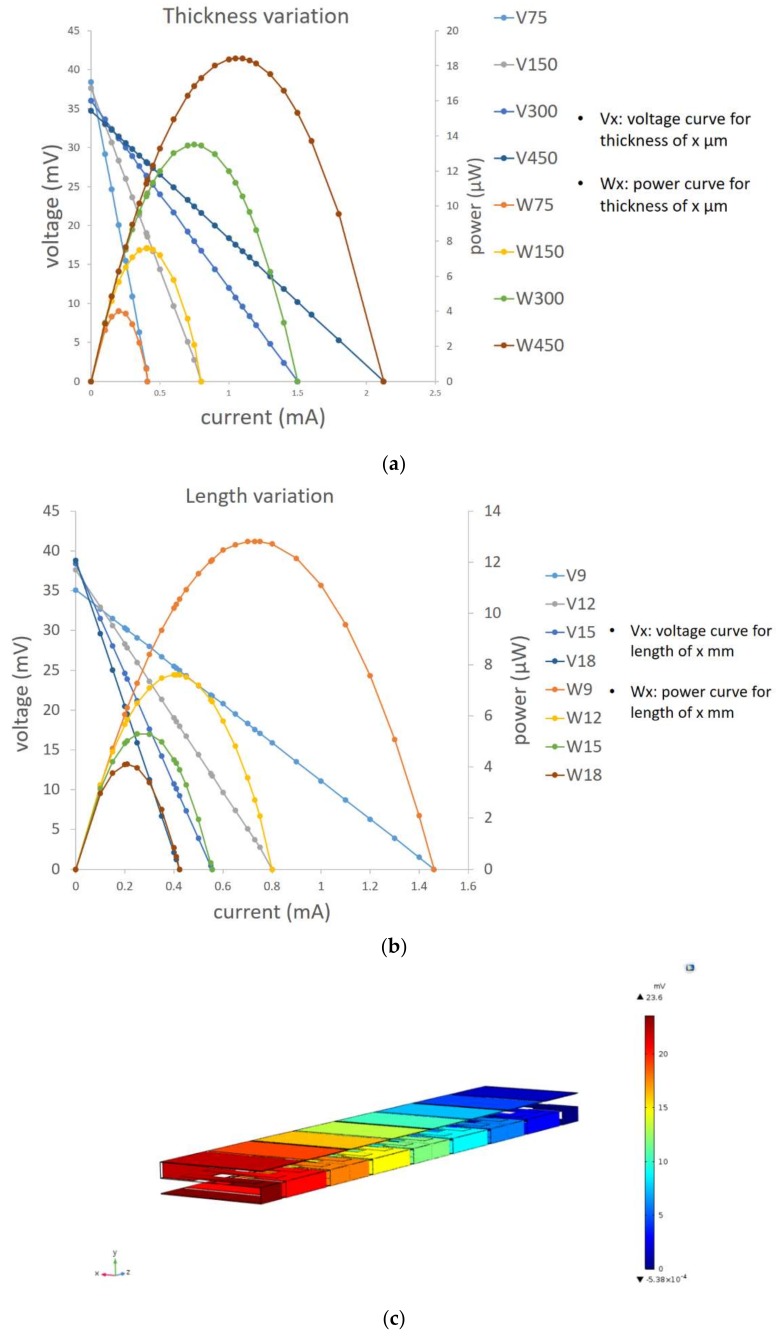
(**a**,**b**) Leg thickness and length variation; (**c**) electrical potential distribution.

According to the maximum power transfer theorem and Equation (1), the maximum power could be reformulated as

(5) $$P_{max}=\;\frac{{\left(V_{o}\right)}^{2}}{4R_{teg}}=\;\frac{{\left[n\left(s_{p}+\;s_{n}\right)\;\Delta T\right]}^{2}}{4n\;\left(r_{p}\frac{L}{A}+r_{n}\frac{L}{A}\right)}$$

where *r_n_* and *r_p_* are the electrical resistivity of the *n*- and *p*-type elements, *A* is the cross-sectional area, and *L* is the length of each leg, respectively. However, in the case of TEGs, a nonlinear behavior, because of the Joule heating and Peltier effect, needs to be considered to find the maximum amount of power. The combination of these alternative situations and parameters are simulated by means of the COMSOL version 5.3 (supported by COMSOL A/S, Lyngby, Denmark) thermoelectric model and will be discussed in this section (Figure 7c shows a typical electrical potential distribution after simulation with COMSOL). Yet, the Equation (5) also would be beneficial to analyze the obtained results. At first, the thickness of TE legs is altered. This happens by parametric 3D modeling of the module in COMSOL version 5.3 (supported by COMSOL A/S, Lyngby, Denmark) and altering only one geometric parameter each time. Based on Equation (5), increasing the thermoelectric legs results in larger cross-sectional area, and eventually increasing the maximum output power. This also complies with the obtained results, which is shown in Figure 7a. There are four alternatives, 75, 150, 300, and 450 μm, for the leg thickness in this simulation. The same comparison is carried out in terms of different thermoelectric lengths, 9, 12, 15, and 18 mm. As shown in Figure 7b, the maximum output power has a decreasing trend with increasing the lengths of the thermoelectric legs, which is also predictable based on the Equation (5) by increasing *R_teg_*. 

**Figure 8:**
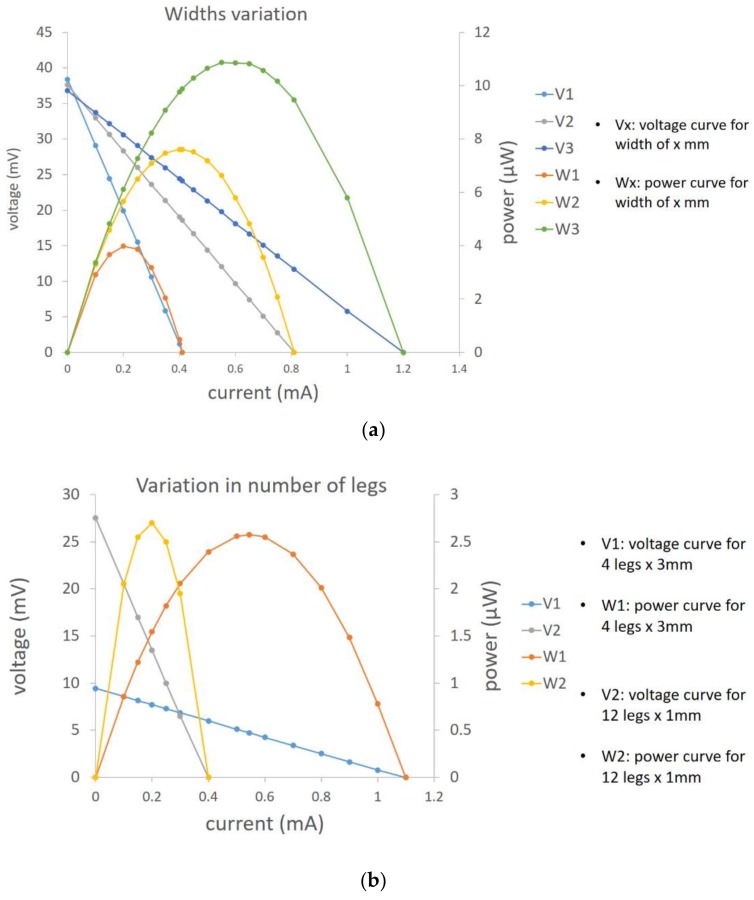
(**a**,**b**) Variation in leg’s widths and number of legs respectively.

For further investigation of the geometric parameters, the thermoelectric widths are altered in two different scenarios. First, it is assumed that the total number of thermocouples is fixed, and then in the second case, the number of thermocouples increases to fill the gap of decreasing the widths (4 legs with width of 3 mm and 12 legs with width of 1 mm). Figure 8a indicates that by increasing the width (1, 2, and 3 mm), the maximum power is also increased, which is confirmed by increasing the amount of legs cross section area in Equation (5). In the second case, there are a couple of parameters that effect the output power in reverse direction (for example, increasing the number of legs leads to a greater open circuit voltage), but on the other hand, the overall internal electrical resistance increases simultaneously, which needs to be considered to find the optimum device configuration for the desired electrical external load. Figure 8b illustrates these dependencies for two various cases in terms of number of thermocouples.

## 6. Conclusions

A new concept for system level optimization and manufacturing of printed organic thin film thermoelectric generators is introduced. It is shown that folding the flexible substrate after printing the thermoelectric materials can retrieve the small amount of thermal gradient for the lateral device configurations. It is also indicated that the concepts can be more customized by means of an FEM tool like COMSOL Multiphysics version 5.3 (supported by COMSOL A/S, Lyngby, Denmark) in terms of the device geometry in order to achieve the best performance with regard to the maximum feasible electrical power. At the end, it is also worth mentioning that a prototype demonstration made by printed thermoelectric legs and interconnects on a kapton substrate is already underway to further proof the concept. Modeling of the different thermal and electrical parasitics will also be a part of the future optimization.

## Figures and Tables

**Table 1:** Values for geometries, material properties, and boundary conditions.

Parameter	Symbol	Value
Seebeck coefficient (p/n-type)	*S_p/n_*	±215 μV/K
Electrical conductivity (p/n-type)	*σ_p/n_*	70 S/cm
Thermal conductivity (p/n-type)	*k_p/n_*	0.37 W/m-K
Hot side temperature	*T_H_*	304.65 K
Ambient temperature	*T_A_*	293.15 K
Natural heat transfer coefficient	*h_A_*	5.46 W/m^2^-K
TE Leg length	*L*	12 mm
TE leg width	*W*	2 mm
TE leg thickness	*T_l_*	150 μm
Copper interconnects thickness	*t_1_*	100 μm
Thickness of Kapton substrate (polyimide)	*T_k_*	25 μm
Thermal conductivity of interconnecters (copper)	*K_cu_*	400 W/m-K
Thermal conductivity of Kapton (polyimide)	*K_s_*	0.12 W/m-K
Electrical conductivity of interconnects	*σ_cu_*	5.998 × 10^7^ S/m
